# Identifying Autophagy-Related lncRNAs and Potential ceRNA Networks in NAFLD

**DOI:** 10.3389/fgene.2022.931928

**Published:** 2022-06-30

**Authors:** Ziyi Cao, Li Guan, RunZhi Yu, Jie Chen

**Affiliations:** Department of Gastroenterology, Huadong Hospital Affiliated to Fudan University, Shanghai, China

**Keywords:** nonalcoholic fatty liver disease, nonalcoholic steatohepatitis, autophagy, ceRNA, lncRNA

## Abstract

Nonalcoholic fatty liver disease (NAFLD) is a common chronic disease with complex pathogenesis, which brings economic burden to the society, and there is still no effective therapy. Impaired autophagy has been implicated in the development of NAFLD. Long noncoding RNAs (lncRNAs) are also reported to play a role in the pathogenesis of NAFLD. However, the role of autophagy-related lncRNAs in NAFLD disease has not been elucidated. Here, we mined GSE135251, GSE160016, GSE130970 and GSE185062 datasets from the Gene Expression Omnibus database (GEO) and obtained the human autophagy-related gene list from the Human Autophagy Database (HADb) for in-depth bioinformatic analysis. Following differential expression analysis and intersection of the datasets, Pearson correlation analysis was performed on DElncRNAs and autophagy-related DEmRNAs to obtain autophagy-related lncRNAs, and then Starbase3.0 and TargetScan7.2 were used to construct competing endogenous RNAs (ceRNA) regulatory networks. We constructed four lncRNA-dominated ceRNA regulatory networks (PSMG3-AS1, MIRLET7BHG, RP11-136K7.2, LINC00925), and visualized with Cytoscape. Then we performed co-expression analysis of the ceRNA networks and autophagy-related genes, and functionally annotated them with Metascape. Finally, we performed receiver operating characteristic curve (ROC) analysis on lncRNAs and mRNAs within the ceRNA networks. Conclusively, our project is the first to study autophagy-related lncRNAs in NAFLD and finally mined four autophagy-related lncRNAs (PSMG3-AS1, MIRLET7BHG, RP11-136K7.2, LINC00925). We suggested that the four autophagy-related lncRNAs may be closely associated with the occurrence and development of NAFLD through the corresponding ceRNA regulatory networks. This research brings new horizons to the study of NAFLD.

## Introduction

Nonalcoholic fatty liver disease (NAFLD) is a well-recognized cause of chronic liver disease, characterized by excess triglyceride (TG) and continuous oxidative stress in hepatocytes (Chu et al., 2019). The NAFLD disease spectrum ranges from simple steatosis, nonalcoholic steatohepatitis, fibrosis to cirrhosis and hepatocellular carcinoma (Eslam et al., 2018). In the US, the number of NAFLD cases is predicted to expand from 83.1 million in 2015 (∼25% of the population) to 100.9 million in 2030, and the proportion of non-alcoholic steatohepatitis (NASH) cases is expected to increase from 20 to 27% of NAFLD adults (Friedman et al., 2018). According to a recent meta-analysis, the prevalence of NAFLD is high in Western and Asian countries, especially China. Accordingly, China is expected to have the largest number of patients with NAFLD and liver-related deaths worldwide (Zhou et al., 2020). The “two-hit” hypothesis is the most well-known theory on the pathogenesis of NAFLD (Buzzetti et al., 2016). The “first hit” is intrahepatic fat accumulation, which leads to the “second hit”, such as inflammation, oxidative stress, and mitochondrial dysfunction, causing hepatocyte injury, apoptosis, and regeneration (Savary et al., 2012). However, this theory cannot fully explain the development of NAFLD. Moreover, the socioeconomic burden associated with this disease is heavy. Indeed, a better understanding of the underlying mechanisms is required to refine the current treatment approach for NAFLD.

Autophagy is a complex molecular pathway that transports intracellular components to the lysosomal compartment for degradation and recycling (Klionsky et al., 2021). Functionally, autophagy is indispensable for maintaining cell homeostasis and is closely related to the pathophysiology of many diseases (Liu et al., 2020). Disturbance of autophagy function can lead to the occurrence and progression of cardiovascular disease, neuropathy, autoimmune disease and malignant tumor (Glick et al., 2010). An increasing body of evidence from recently published studies suggests that autophagy is also closely related to NAFLD disease. Autophagy plays a fundamental role in lipid droplet turnover, and inhibition of this process can cause increased lipid droplet content in hepatocytes cultured in the basal state or exposure to free fatty acids (Singh et al., 2009). Besides, autophagy is involved in the selective packaging and breakdown of glycogen particles into glucose (Ueno and Komatsu, 2017), which leads to insulin resistance when inhibited (Yang et al., 2010). Furthermore, autophagy inhibition is associated with liver inflammation and endoplasmic reticulum stress (Yang et al., 2010). Thus, abnormal hepatic autophagy participates in the occurrence and development of NAFLD.

Long noncoding RNAs refer to a kind of ncRNAs longer than 200 nucleotides transcribed by RNA polymerase II (Chen, 2016). Although most lncRNAs are lowly expressed compared with protein-coding genes, they exhibit specific regulatory roles in different tissues, probably due to tissue-specific and developmental-stage specific expression patterns (Wong et al., 2018). The number of functional lncRNAs remains subject to debate. Although the functions of a large number of lncRNAs remain unexplored, there is ample evidence that lncRNAs have important cellular functions (Statello et al., 2021). Aberrant expression of specific lncRNAs is associated with the development of human diseases of diverse systems, including the nervous, cardiovascular, and immune systems (Fatica and Bozzoni, 2014; Statello et al., 2021). Growing evidence suggests that lncRNAs participate in NAFLD progression and contribute to diagnosis as molecular biomarkers (Fatica and Bozzoni, 2014; Zhao et al., 2018). LncLSTR is highly expressed in the liver and upregulates apoC2 expression, initiating LPL activation and enhancing serum triglycerides clearance (Lonardo et al., 2015). LncRNA Gm16551 can inhibit *de novo* lipogenesis at low expression levels, while lncRNA H19 promotes fibrogenesis at high expression levels in NAFLD (Di Mauro et al., 2021). Moreover, lncRNA HCG18 affects fat deposition and glucose disorder in NAFLD (Xia et al., 2021a).

Overwhelming evidence substantiates the relationship between lncRNAs and autophagy, which has been explored in tumors, cardiovascular disease and other systemic diseases (Gupta et al., 2021; Li et al., 2021; Zhou et al., 2021). However, it remains unclear whether autophagy-related lncRNAs participate in NAFLD. In this study, we screened four autophagy-related lncRNAs from datasets integrated from the Gene Expression Omnibus (GEO) database and HADb database and established putative lncRNA-miRNA-mRNA regulatory networks. Overall, our results showed that lncRNA PSMG3-AS1, MIRLET7BHG, RP11-136K7.2 and LINC00925 might play a role in NAFLD, providing new insights and the foothold for further studies on mechanism exploration of NAFLD.

## Materials and Methods

### Data Acquisition

NAFLD gene expression profile (GSE135251) comprising 206 NAFLD cases with different fibrosis stages and 10 controls were obtained from the Gene Expression Omnibus (GEO) database (https://www.ncbi.nlm.nih.gov/geo/). The GSE160016 (6 NAFLD cases and five control cases) and GSE130970 (6 histologically normal cases and 72 NAFLD diseases covering the full disease spectrum) datasets were downloaded. GSE185062, which contains the peripheral-blood miRNA expression profile of 183 NAFLD cases representing the complete NAFLD spectrum and 10 population controls, was also downloaded.

### Data Processing

First, we analyzed the gene expression profiles in GSE135251, GSE160016, and GSE130970 to acquire lncRNA expression matrixes and mRNA expression matrixes by the file Homo_sapiens.GRCh37.87. chr.gtf on the Ensembl website (http://asia.ensembl.org). Next, lncRNA expression matrixes from GSE135251 and GSE160016, mRNA expression matrixes from GSE135251 and GSE130970 and miRNA expression matrix from GSE185062 were used to perform differential expression analysis by the limma package in R software. Differentially expressed genes were identified using the following criteria: |log2 fold change (FC)| > 0.58 or |FC|>1.5 and *p* < 0.05. DEmiRNAs were identified from GSE185062. The intersection of differentially expressed lncRNAs between GSE135251 and GSE160016 was used to obtain DElncRNAs. Target DEmRNAs were obtained by intersecting differentially expressed mRNAs between GSE135251 and GSE130970. An autophagy-related genes list was downloaded from the Human Autophagy (HADb) Database (http://www.autophagy.lu/). By intersecting autophagy genes with target differentially expressed mRNAs, we obtained a group of differentially expressed autophagy-related genes (autophagy-related DEmRNAs).

### GO and KEGG Analyses of Autophagy-Related DEmRNAs

The R package cluster profiler was used to conduct Gene Ontology (GO) and Kyoto Encyclopedia of Genes and Genomes analyses (KEGG) of autophagy-related DEmRNAs. A *p*-value <0.05 was statistically significant. The GO and KEGG results were visualized by the ggplot2 package. Only the top 20 results were shown.

### Identification of Autophagy-Related lncRNAs

Pearson correlation analysis was conducted using R software to analyze correlations between target differentially expressed lncRNAs and autophagy-related DEmRNAs in the GSE135251 NAFLD gene expression profile. |r| > 0.4 and *p* < 0.05 were set as the thresholds to screen autophagy-related lncRNAs in NAFLD.

### Construction of the lncRNA-miRNA-mRNA Regulatory Network

To construct ceRNA regulation networks, interactions between autophagy-related lncRNAs and DEmiRNAs were predicted on the StarBase 3.0 website (http://starbase. sysu. edu.cn/). Then the DEmiRNAs in these interaction pairs were used to identify corresponding mRNAs that potentially bind to them, based on the StarBase 3.0 website and TargetScan database (version: release 7.2, http://www.targetscan.org/). The intersection of predicted target mRNAs with differentially expressed autophagy-related genes in NAFLD yielded candidate target mRNAs in the ceRNA network. The potential lncRNA-miRNA-mRNA regulatory networks were visualized using Cytoscape3.8.2.

### Functional Analysis

After establishing the ceRNA network, Pearson correlation analysis was used to analyze the co-expression relationship between the ceRNA networks and autophagy-related genes, and the threshold was set to |r| >0.4 and *p* < 0.05. Then, we performed a functional analysis of these genes using the Metascape annotation tool.

### Receiver Operator Characteristic Curve Analysis

Receiver operator characteristic curve (ROC) analysis was applied to verify the diagnostic accuracy of autophagy-related lncRNAs and autophagy-related DEmRNAs. The GSE160016 and GSE130970 datasets were used to assess the diagnostic performance of the screened lncRNAs and autophagy-related mRNAs, respectively, in NAFLD disease, in terms of the area under the receiver operator characteristic curve (AUC) values.

## Result

### DElncRNAs and DEmRNAs in NAFLD

**FIGURE 1:**
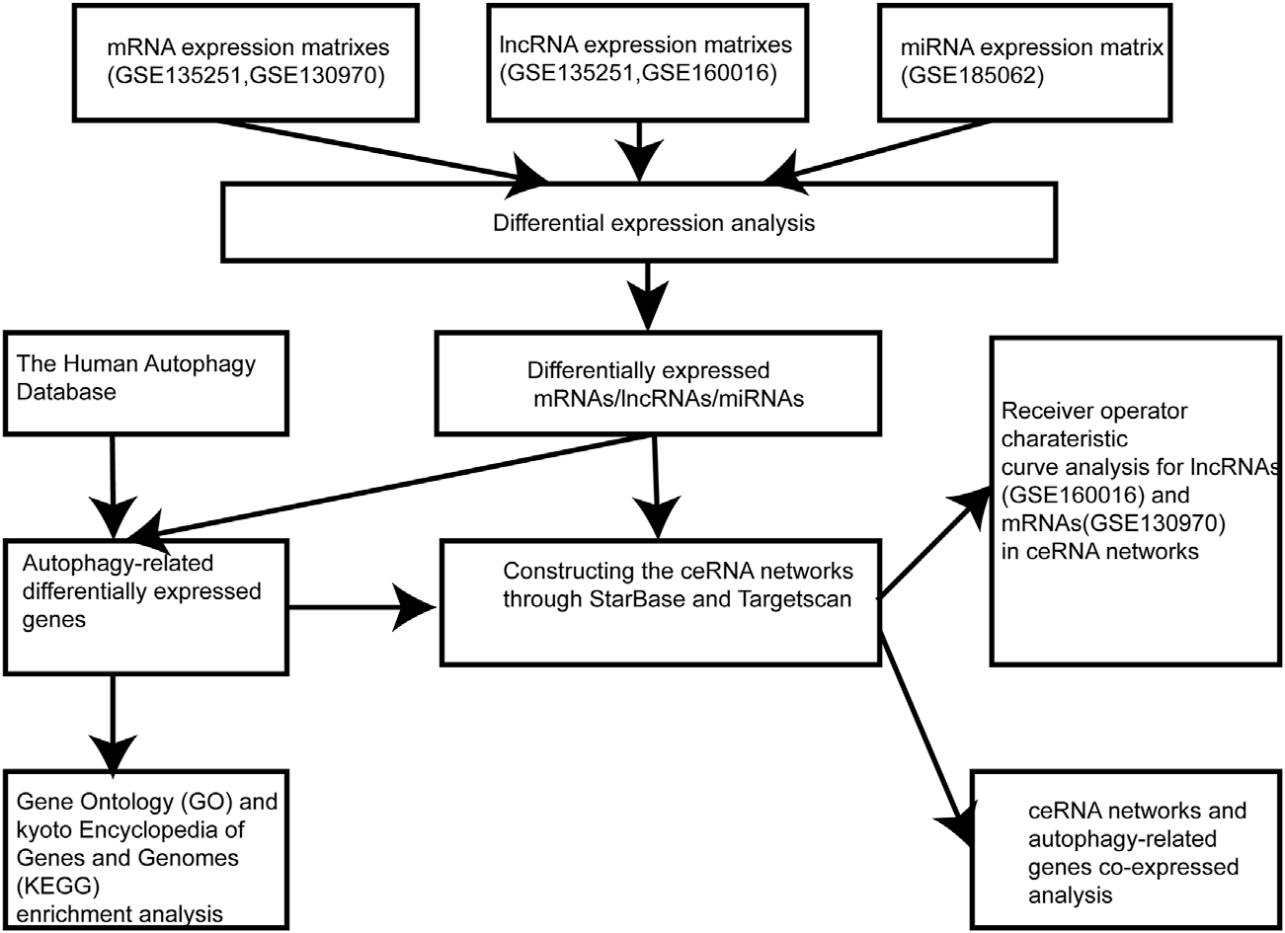
Flowchart of the research design. lncRNA, long noncoding RNA; miRNA, microRNA.

**FIGURE 2:**
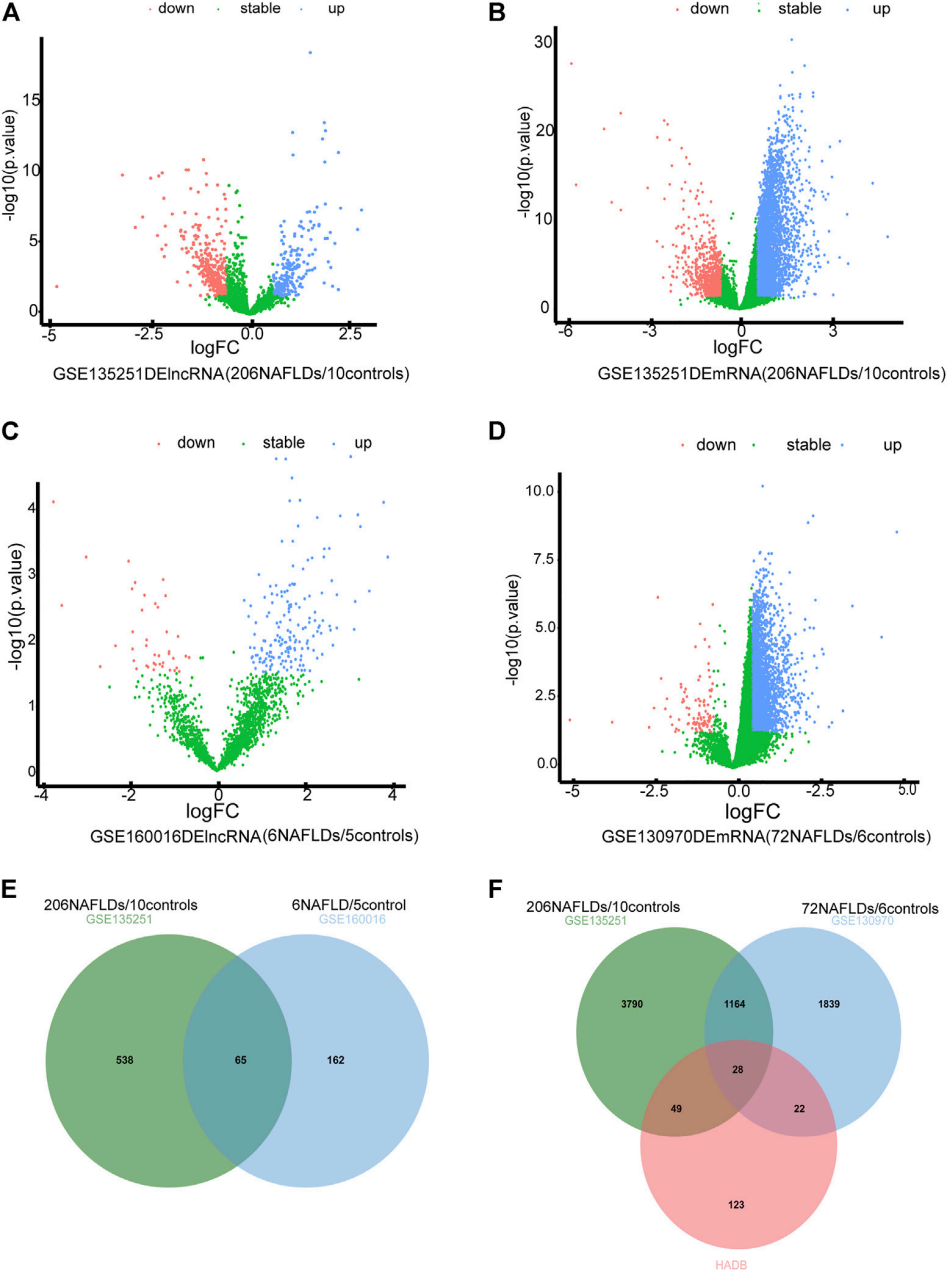
Differentially expressed autophagy-related genes and lncRNAs. **(A)** volcano plot of DElncRNAs in GSE135251 (|log2 fold change| >0.58; *p* < 0.05) **(B)** volcano plot of DEmRNAs in GSE135251 (|log2 fold change| >0.58; *p* < 0.05). **(C)** volcano plot of DElncRNAs in GSE160016 (|log2 fold change| >0.58; *p* < 0.05). **(D)** volcano plot of DEmRNAs in GSE130970 (|log2 fold change| >0.58; *p* < 0.05). **(E)** Intersection of DElncRNAs of GSE135251 and GSE160016. **(F)** Intersection of DEmRNAs of GSE135251 and GSE130970 with autophagy genes of HADb.

The flowchart of the research design was shown in Figure 1. In the GSE135251 dataset, 603 differentially expressed lncRNAs were found compared to NAFLD and control groups, including 258 upregulated lncRNAs and 345 downregulated lncRNAs (Figure 2A). 5,031 differentially expressed mRNAs were also identified in this dataset, containing 4,104 upregulated and 927 downregulated mRNAs (Figure 2B). As shown in figure 2C, 237 differentially expressed lncRNAs were selected in the GSE160016 dataset, among which 194 were upregulated and 43 were downregulated. After analysis of the GSE130970 dataset, 3,058 differentially expressed mRNAs were selected, of which 2,959 mRNAs were upregulated, and 99 mRNAs were downregulated (Figure 2D). We used the differentially expressed lncRNAs (*n* = 65) between GSE135251 and GSE160016 as the target DElncRNAs for subsequent analysis (Figure 2E). The Human Autophagy (HADb) database contained 232 human autophagy-related genes. Finally, 28 differentially expressed mRNAs among GSE135251, GSE130970 datasets and HADb were identified as autophagy-related DEmRNAs in NAFLD (Figure 2F).

### Functional Enrichment Analysis of Autophagy-Related DEmRNAs in NAFLD

**FIGURE 3:**
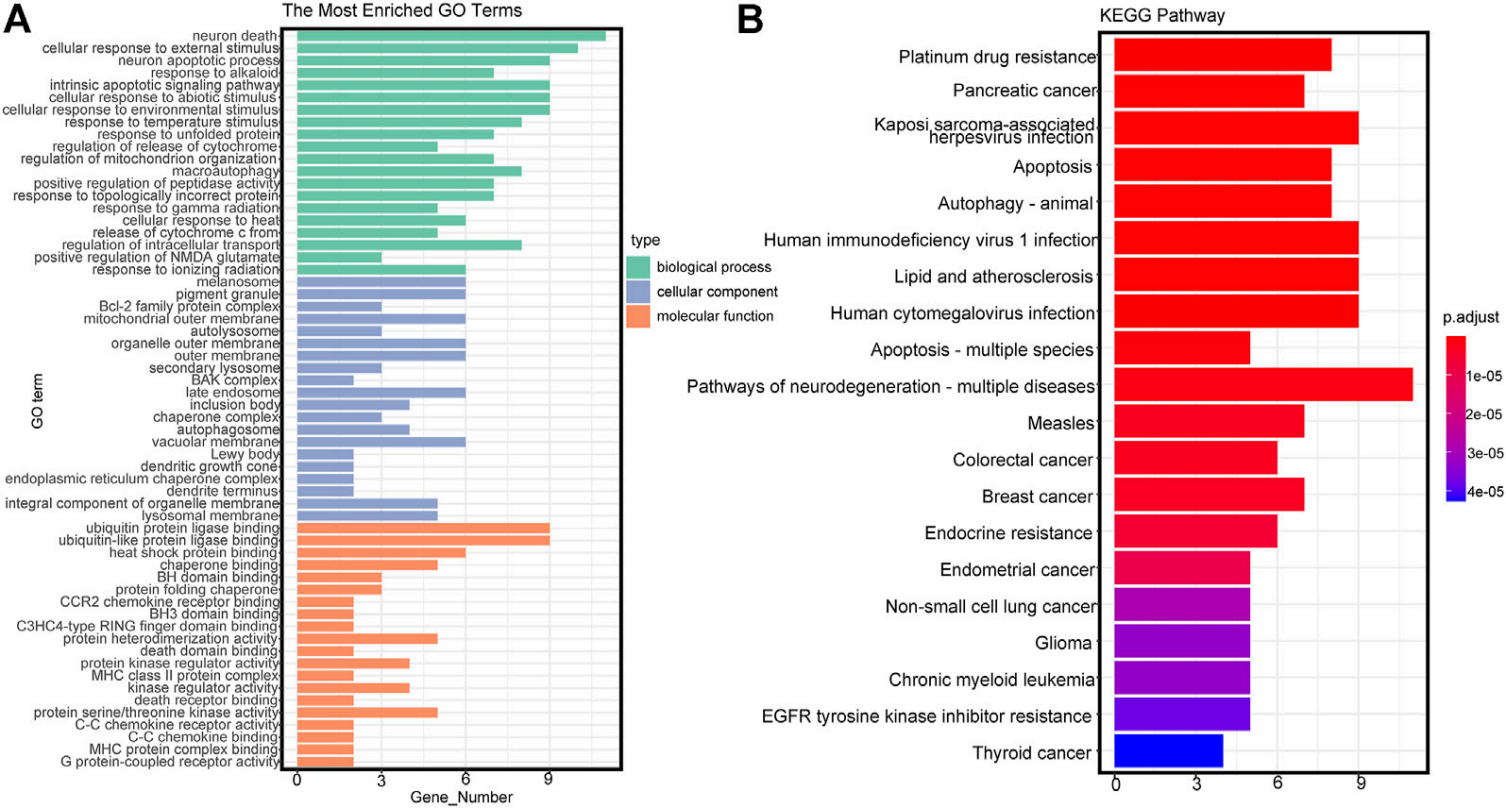
Gene Ontology (GO) and Kyoto Encyclopedia of Genes and Genomes (KEGG) terms of differentially expressed autophagy-related genes. **(A)** GO analysis based on differentially expressed autophagy-related genes. BP, biological process; CC, cellular component; MF, molecular function. **(B)** KEGG analysis based on differentially expressed autophagy-related genes.

Twenty-eight autophagy-related DEmRNAs were assessed by cluster analysis and visualized by ggplot2. As shown in Figure 3A, GO analysis of the 28 autophagy-related genes showed significant enrichment in biological functions (BPs) associated with response to neuron death, cellular response to external stimulus, intrinsic apoptotic signaling pathway, etc. Moreover, the cellular component (CCs) of these mRNAs included the Bcl-2 family protein complex, BAX complex, autolysosome, etc. Besides, the significantly enriched molecular function terms were associated with ubiquitin-like protein ligase binding, heat shock protein binding, chaperone binding and so on. KEGG pathway enrichment analysis of autophagy-related genes showed significant enrichment in EGFR tyrosine kinase inhibitor resistance, endocrine resistance, lipid and atherosclerosis, etc. (Figure 3B).

### Identification Autophagy-Related lncRNAs and DEmiRNAs in NAFLD

**FIGURE 4:**
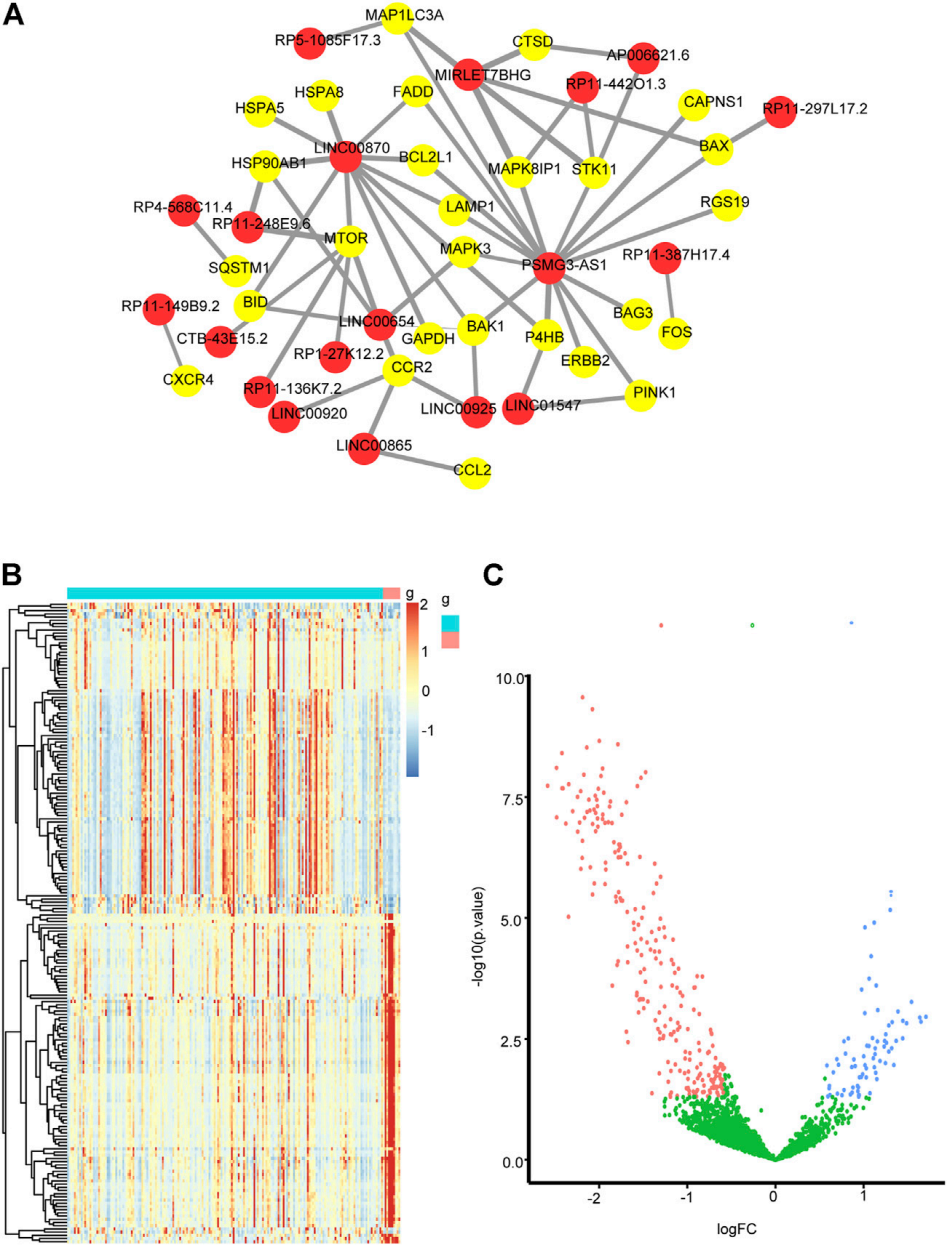
**(A)** Regulatory networks between autophagy-related lncRNAs and autophagy-related genes. The red dots mark lncRNAs, the yellow dots mark autophagy-related genes (|r|>0.4, *p* < 0.05). **(B)** a heatmap of DEmiRNAs in GSE185062 (|log2 fold change| >0.58; *p* < 0.05) and **(C)** a volcano plot of DEmiRNAs in GSE185062 (|log2 fold change| >0.58; *p* < 0.05).

Pearson correlation analysis was conducted on autophagy-related DEmRNAs and DElncRNAs to identify autophagy-related lncRNAs. A total of 19 autophagy-related lncRNAs were selected. The autophagy-related lncRNA−mRNA regulatory network is shown in Figure 4A. In the GSE185062 dataset, 320 differential miRNAs were found between NAFLD and control groups, including 74 upregulated miRNAs and 246 downregulated miRNAs (Figures 4B, C).

### Construction of the ceRNA Regulatory Network

**FIGURE 5:**
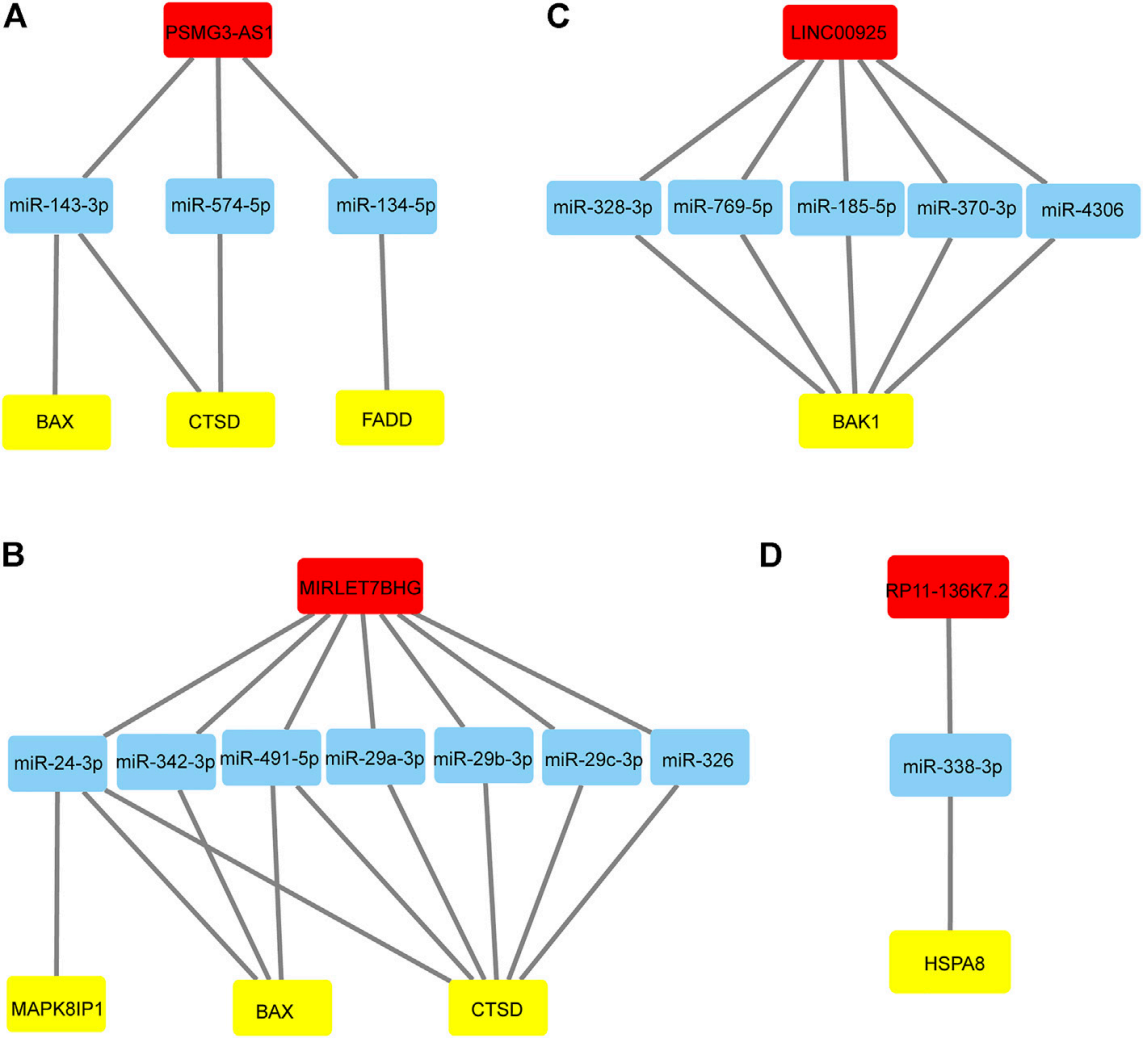
lncRNA-miRNA-mRNA regulatory networks **(A)** LncRNA PSMG3-AS1 and its binding miRNAs and the corresponding regulation of autophagy-related genes. **(B)** LncRNA MIRLET7BHG and its binding miRNAs and the corresponding regulation of autophagy-related genes. **(C)** LncRNA LINC00925 and its binding miRNAs and the corresponding regulation of autophagy-related genes. **(D)** LncRNA RP11-136K7.2 and its binding miRNAs and the corresponding regulation of autophagy-related genes.

Given that miRNA negatively regulates target RNA molecules, when the expression of lncRNAs is increased, lncRNAs may bind more miRNAs, thereby weakening the negative regulation of miRNAs on mRNAs, leading to increased mRNA expression. Therefore, we selected autophagy-related lncRNAs and autophagy-related DEmRNAs with positive regulatory relationships and DEmiRNAs to construct ceRNA networks through Starbase3.0 and TargestScan7.2. We finally constructed four lncRNA-dominated ceRNA networks. As shown in Figure 5A, lncRNA PSMG3-AS1 regulated CTSD, BAX and FADD by interacting with miR-143-3p, miR-574-5p and miR-134-5p. MIRLET7BHG regulated MAPK8IP1, BAX and CTSD through binding to miR-24-3p, miR-342-3p, miR-491-5p, miR-326, miR-29a -3p, miR-29b-3p, and miR-29c-3p (Figure 5B). LINC00925 interacted with five miRNAs to regulate BAK1, including miR-185-5p, miR-370-3p, miR-328-3p, miR-769-5p, miR-4306 (Figure 5C). As shown in Figure 5D, lncRNA RP11-136K7.2 interacted with miR-338-3p to affect the expression level of HSPA8. Interestingly, PSMG3-AS1 and MIRLET7BHG regulated CTSD and BAX, suggesting they may perform similar functions in NALFD.

### Functional Analyses of the ceRNA Network

**FIGURE 6:**
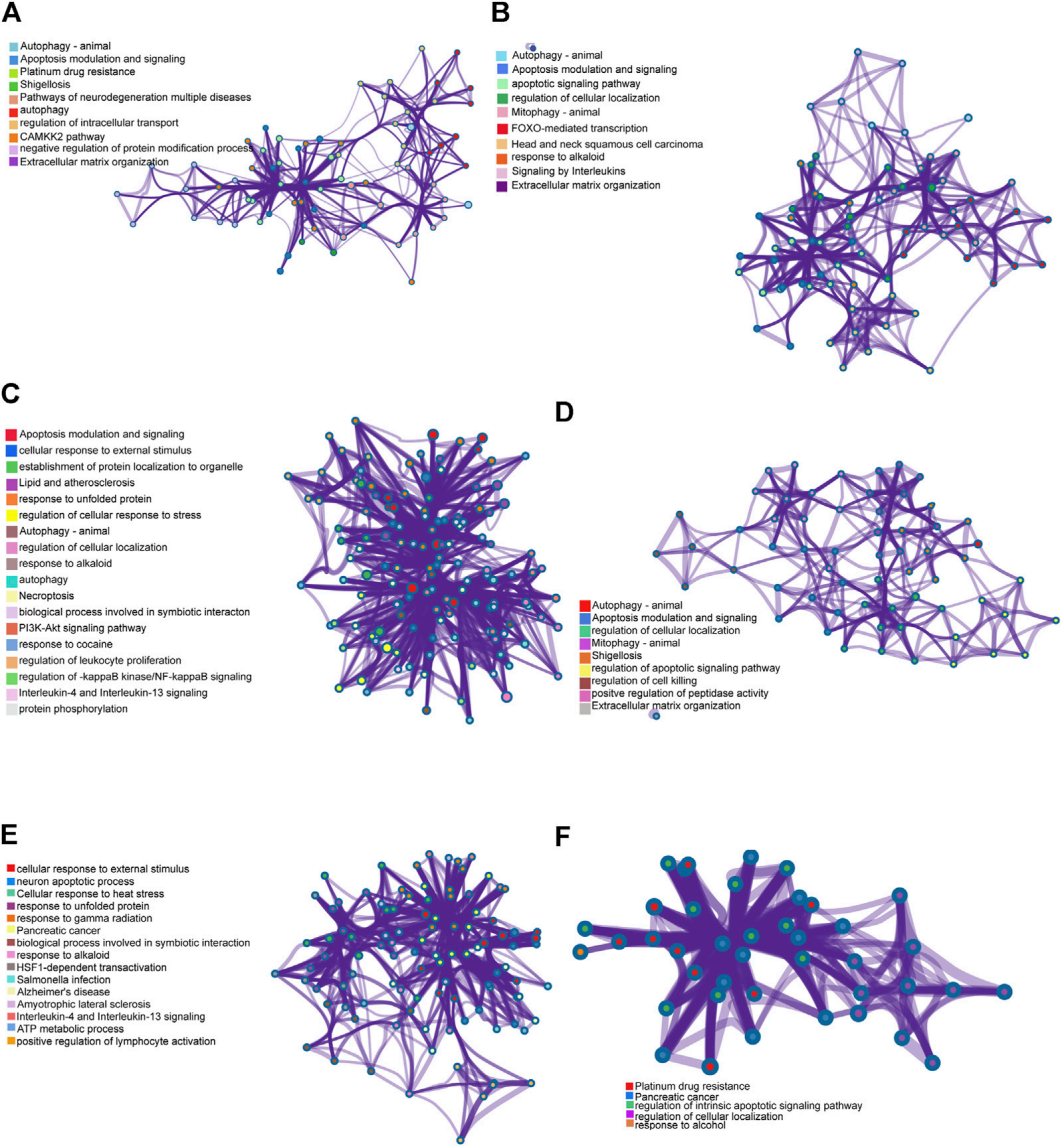
Genes of co-expression network and their enrichment analyses. **(A)** Functional annotation of CTSD and its co-expressed genes. **(B)** Functional annotation of BAX and its co-expressed genes. **(C)** Functional annotation of FADD and its co-expressed genes. **(D)** Functional annotation of MAPK8IP1 and its co-expressed genes. **(E)** Functional annotation of HSPA8 and its co-expressed genes. **(F)** Functional annotation of BAK1 and its co-expressed genes.

Pearson correlation analysis was performed between autophagy-related genes and ceRNAs to detect co-expression relationships, following the construction of the ceRNA networks of the lncRNA− miRNA−mRNA networks. Six co-expressed genes were explored: CTSD, BAX, FADD, MAPK8IP1, HSPA8 and BAK1. Metascape was used to perform functional annotation. Functional annotation of CTSD and its co-expressed genes showed that lncRNA PSMG3-AS1 and MIRLET7BHG in NAFLD controlled the expression of CTSD through their target miRNAs, subsequently regulating the CAMKK2 pathway, extracellular matrix organization, autophagy, etc. (Figure 6A). Functional annotation of BAX and its co-expressed genes suggested that lncRNA PSMG3-AS1 and MIRLET7BHG in NAFLD may regulate the expression of BAX, leading to regulation of FOXO-mediated transcription, apoptotic signaling pathway, and mitophagy and so on (Figure 6B). Functional annotation of FADD and its co-expressed genes showed that PSMG3-AS1 might control the expression of FADD by miR-134-5p in NAFLD, thereby regulating the PI3K-AKT signaling pathway, IL-4 and IL-3 signaling, and NF-kappaB signaling (Figure 6C). Functional annotation of MAPK8IP1 and its co-expressed genes suggested that MIRLET7BHG may mediate MAPK8IP1 expression through binding to miR-24-3p, thereby regulating cell killing, cellular localization, and peptidase activity (Figure 6D). As shown in Figure 6E, functional annotation of HSPA8 and its co-expressed genes suggested that RP11-136K7.2 may affect the expression of HSPA8 through miR-338-3p to regulate ATP metabolic process, cellular response to stimulus, IL-4 and IL-13 signaling, etc. Functional annotation of BAK1 and its co-expressed genes suggested that LINC00925 may regulate the expression of BAK1 through miR-185-5p, miR-370-3p, miR-328-3p, miR-769-5p, and miR-4306, thereby regulating the cellular localization, intrinsic apoptotic signaling pathway, and alcohol response (Figure 6F).

### Diagnostic Value of the Four Autophagy-Related lncRNAs and Their Corresponding Autophagy-Related Genes

**FIGURE 7:**
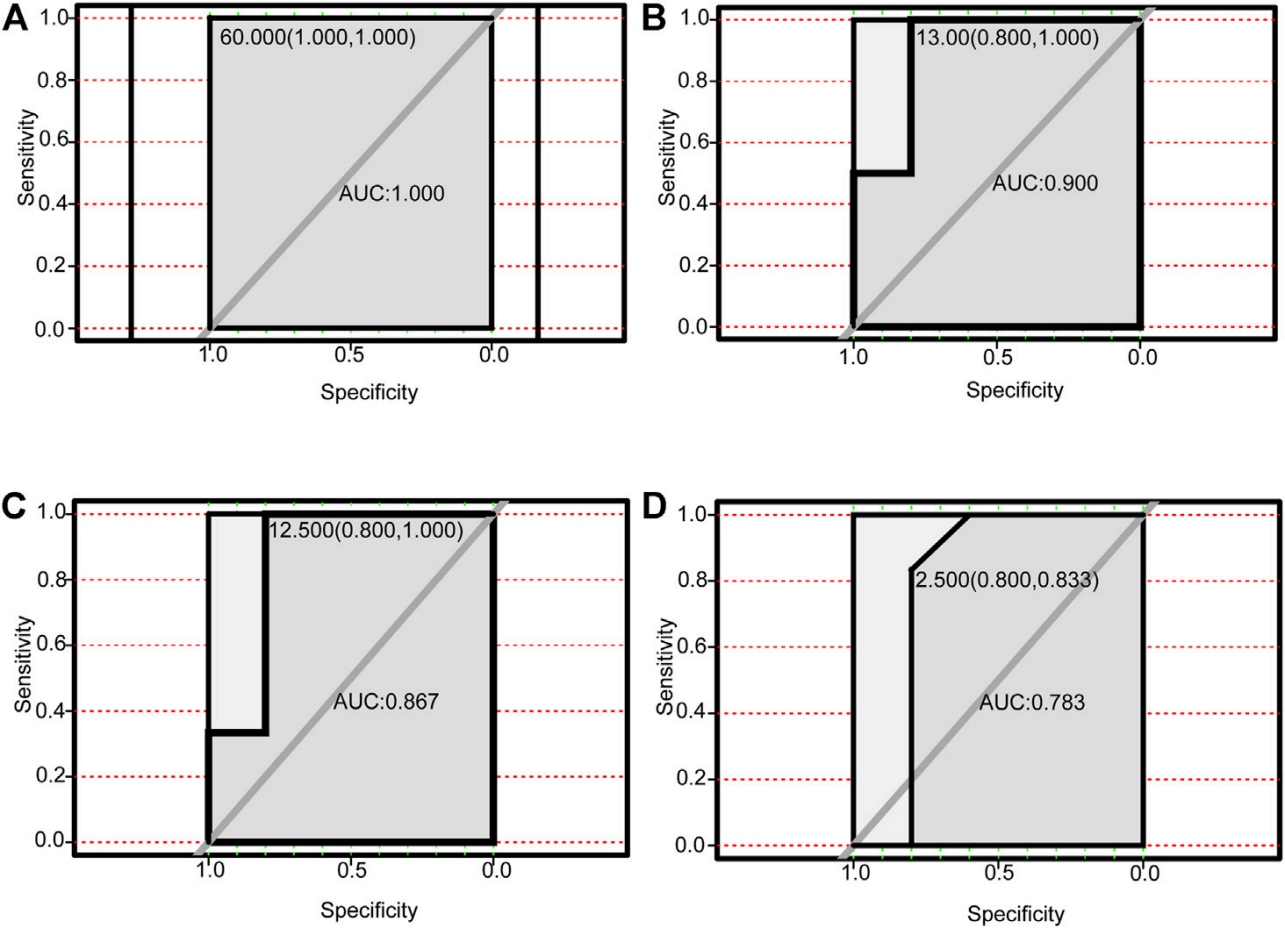
Performance of autophagy-related DElncRNAs for diagnosing NAFLD (GSE160016). **(A)** ROC analysis of PSMG3-AS1. **(B)** ROC analysis of MIRLET7BHG. **(C)** ROC analysis of RP11-136K7.2. **(D)** ROC analysis of LINC00925.

**FIGURE 8:**
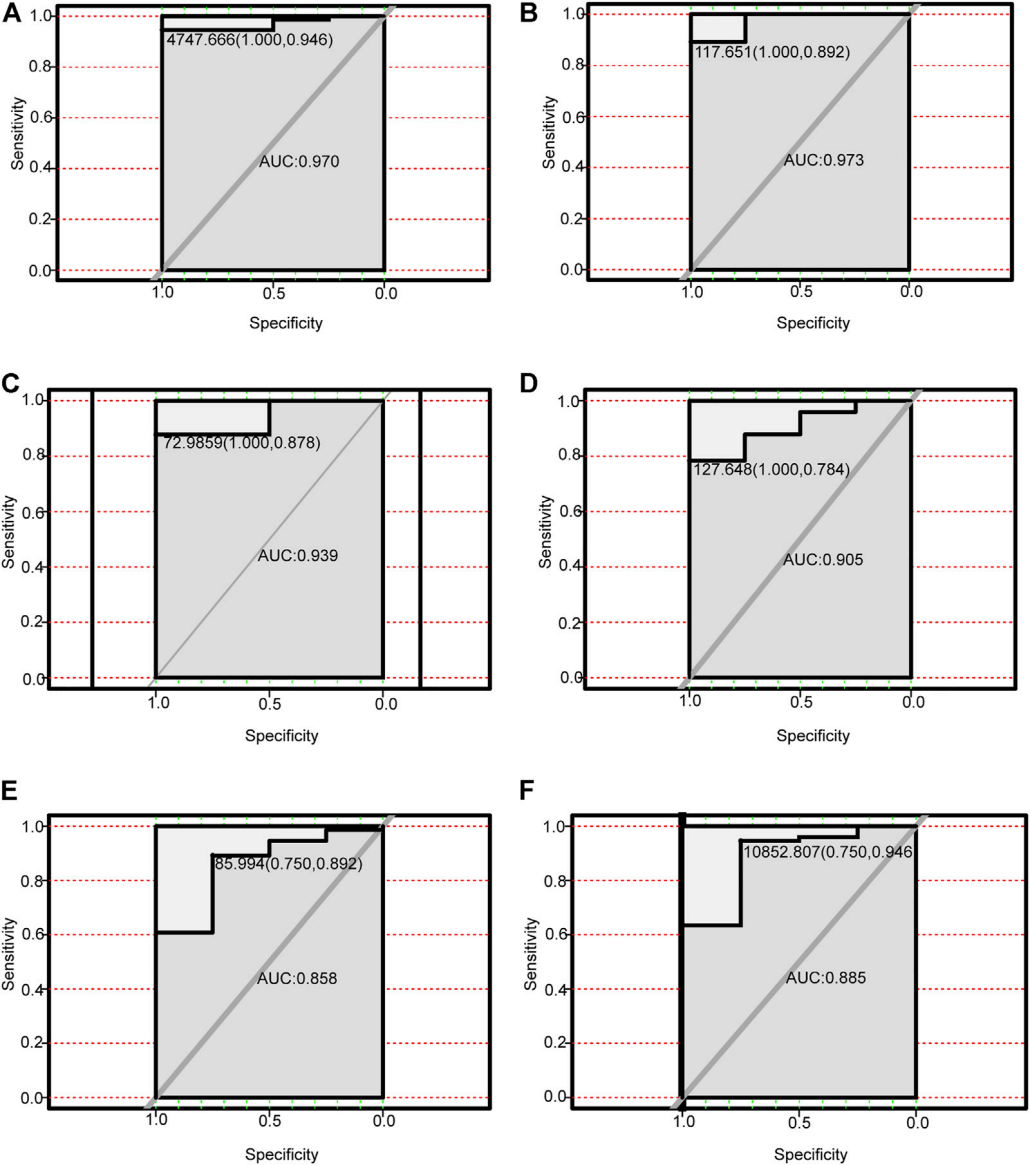
Performance of autophagy-related DEmRNAs for diagnosing NAFLD (GSE130970). **(A)** ROC analysis of CTSD. **(B)** ROC analysis of FADD. **(C)** ROC analysis of BAK1. **(D)** ROC analysis of BAX. **(E)** ROC analysis of MAPK8IP1. **(F)** ROC analysis of HSPA8.

We performed receiver operating characteristic curve (ROC) analysis on the four lncRNAs and the corresponding six autophagy-related genes within the ceRNA networks. The core lncRNAs exhibited excellent ability to differentiate NAFLD patients from controls in GSE160016. Among them, PSMG3-AS1 yielded the best diagnostic performance (AUC = 1.0 Figure 7A), followed by MIRLET7BHG (AUC = 0.9, Figure 7B), RP11-136K7.2 (AUC = 0.87, Figure 7C), LINC00925 (AUC = 0.78, Figure 7D). Overall, the six autophagy-related genes also yielded good diagnostic value for NAFLD in GSE130970. CTSD (AUC = 0.97) and FADD (AUC = 0.97) exhibited the best performance (Figures 8A, B), followed by BAK1 and BAX (AUC = 0.94 and 0.91, respectively, Figures 8C, D). Among the six genes, MAPK8IP1and HSPA8 had the poorest diagnostic value (AUC = 0.86 and 0.89, respectively, Figures 8E, F).

## Discussion

NAFLD consists of a wide spectrum of diseases, ranging from benign prognostic steatosis to liver fibrosis, cirrhosis, and even cancer (Marrero et al., 2002). NAFLD is widely thought to result from the interplay of genetic, environmental and metabolism; however, the exact mechanisms remain to be elucidated. Accordingly, there is an urgent need to study the potential molecular mechanisms of NALFD and provide novel ideas for clinical treatment.

Autophagy is a ubiquitous cellular homeostasis program (Klionsky and Emr, 2000) and an adaptive response to both intracellular and extracellular stress, such as endoplasmic reticulum stress, damaged cellular organelles and proteins or starvation (Lum et al., 2005; González-Rodríguez et al., 2014). In recent years, autophagy has been linked to the development of NAFLD since it can reduce cellular lipid accumulation (Singh et al., 2009), inflammatory response (Kim and Kim, 2020) and insulin resistance (Ueno and Komatsu, 2017). Moreover, alterations in hepatic autophagy have been detected in experimental animal models of nonalcoholic and alcoholic hepatic steatosis (Yang et al., 2010; Ezquerro et al., 2016).

Over the past few years, significant inroads have been made in our understanding of the role of lncRNAs. The various functions of lncRNAs affect different aspects of physiology, such as cell cycle regulation, cell differentiation, gene post-transcriptional regulation and responses to diverse stresses and stimuli (Chen et al., 2019; Zottel et al., 2020; Ren et al., 2021). LncRNAs can reportedly compete for miRNA binding, thereby modulating the expression of genes. Importantly, lncRNAs, miRNAs and mRNAs can form a ceRNA regulatory network to affect various physical activities of cells (Rohilla et al., 2021). Competing endogenous RNAs (ceRNA) are transcripts that cross-regulate each other by competing for shared microRNAs (miRNAs) (Salmena et al., 2011). Current evidence suggests that some lncRNAs can regulate the expression of some autophagy-related genes. For example, the H19/let 7/Lin28 ceRNA network is involved in autophagy and EMT in breast cancer (BC) (Xiong et al., 2020). LncRNA 2810403D21Rik/Mirf promotes ischemic myocardial injury by regulating autophagy targeting Mir26a (Liang et al., 2020). Moreover, it has been shown that long noncoding RNA SNHG1 activates autophagy and promotes cell invasion in bladder cancer (Guo et al., 2021). It has been shown that thousands of lncRNAs are differentially expressed in NAFLD; however, only a few have been studied, and their association with autophagy genes in NAFLD remains poorly understood. Herein, we sought to mine autophagy-related lncRNAs in NAFLD and construct ceRNA regulatory networks to provide new horizons for an in-depth study of the pathogenesis of NAFLD.

After combining the GEO datasets and the HADb database, we found 28 differentially expressed autophagy-related genes, 65 differentially expressed lncRNAs, and 320 differentially expressed miRNAs in NAFLD. Then, we performed GO and KEGG analysis on autophagy-related DEmRNAs. 19 autophagy-related lncRNAs were identified by Pearson correlation analysis. Four lncRNA-dominated ceRNA networks were subsequently constructed using autophagy-related lncRNAs, autophagy-related DEmRNAs and DEmiRNAs. Moreover, the co-expression analysis of ceRNA networks and autophagy-related genes was performed, and the functional analysis of each co-expression network associated with the occurrence and development of NAFLD was performed using the metascape annotation tool. Finally, four autophagy-related lncRNAs (PSMG3-AS1, MIRLET7BHG, RP11-136K7.2, LINC00925) and corresponding mRNAs (CTSD, BAX, FADD, MAPK8IP1, HSPA8, BAK1) underwent ROC analysis to examine their diagnostic performance for NAFLD.

LncRNA PSMG3-AS1 has been documented in various tumors. Interestingly, overexpression of lncRNA PSMG3-AS1 can distinguish glioblastomas from sarcoidosis and reduce the inhibitory effects of miR-34a on GBM cell proliferation (Chen et al., 2020). Moreover, it has been shown that overexpression of PSMG3-AS1 could increase cervical squamous cell carcinoma cell invasion and migration, which miR-441738 inhibits (Man et al., 2021). Besides, MiR-449b-5p targets lncRNA PSMG3-AS1 to suppress cancer cell proliferation in lung adenocarcinoma (Yue et al., 2020). In addition, PSMG3-AS1 was found to be upregulated in lung squamous cell carcinoma (LUSC) and associated with miR-143-3p (Jin et al., 2021). Indeed, the relationship between PSMG3-AS1 and miR-143-3p has been established in lung cancer, breast cancer and hepatocellular carcinoma. In this regard, the knockdown of PSMG3 AS1 increased miR 143 3p expression, which mitigated the proliferation and migration capacity in breast carcinoma cells and reduced the mRNA and protein expression levels of COL1A1 (Cui et al., 2020). In hepatocellular carcinoma, the expression of PSMG3 AS1 and miR-143-3p were closely and inversely correlated. High expression levels of PSMG3 AS1 predicted poor survival and increased proliferation rates (Zhang et al., 2020). In our study, PSMG3 AS1 was highly expressed in NAFLD and was predicted to bind miR-143-3p, miR-574-5p, miR-134-5p to affect the expression of BAX, CTSD and FADD, which are autophagy-related genes, involved in the pathogenesis and progression of NAFLD. Bax is the most important apoptosis gene in the human body and belongs to the bcl-2 gene family (Lebeaupin et al., 2018). Intriguingly, Bax inhibitor-1, a negative regulator of the ER stress sensor, protects from nonalcoholic steatohepatitis by limiting inositol-requiring enzyme one alpha signaling in mice (Lebeaupin et al., 2018). Recent evidence has indicated that cathepsin D (CTSD), a lysosomal enzyme, is a key player in developing hepatic inflammation and dyslipidemia and is a marker for NASH (Houben et al., 2017). Targeting CASP8 and FADD-like apoptosis regulator ameliorates nonalcoholic steatohepatitis in mice and nonhuman primates (Wang et al., 2017). There is currently no reports on the role of PSMG3 AS1 in NAFLD, although NAFLD progression can lead to liver cancer, and PSMG3 AS1 plays a role in liver cancer. Accordingly, we hypothesized that PSMG3 AS1 is associated with NAFLD by regulating BAX, CTSD and FADD. Moreover, its diagnostic capability for NAFLD (AUC = 1.0) provided further evidence. LncRNA MIRLET7BHG has also been reported to be related to tumor diseases. MIRLET7BHG has been reported as an autophagy-related lncRNA in Acute Myeloid Leukemia (AML) and is associated with poorer survival in patients with high expression levels (Zhao et al., 2021). Interestingly, MIRLET7BHG polymorphisms may be important predictive markers for asbestos exposure-related lung cancer (Liu et al., 2015). Moreover, the role of MIRLET7BHG in genomic instability and cancer prognosis has been studied to provide new insights into the prognostic prediction of colon cancer (Chen et al., 2021). In hepatocellular carcinoma, MIRLET7BHG promotes hepatocellular carcinoma progression by activating hepatic stellate cells through exosomal SMO to trigger the Hedgehog pathway (Xia et al., 2021b). One study has linked MIRLET7BHG to metabolism. Neonatal methylation markers MIRLET7BHG are significantly associated with birth weight and have been previously implicated in metabolic pathways in human and animal models (Lin et al., 2017). In our research, we found that MIRLET7BHG could interact with miR-24-3p, miR-342-3p, miR-491-5p, miR-326, miR-29a -3p, miR-29b-3p, and miR-29c-3p to target CTSD, BAX, and MAPK8IP1. The functions of CTSD and BAX in NAFLD have been elucidated as above. Although no literature has reported MAPK8IP1’s role in NAFLD, it is widely believed to be closely related to metabolism (Waeber et al., 2000). The functions of LINC00925 and RP11-136K7.2 remain unclear. In this study, we found that LINC00925 could target gene BAK1 by regulating miR-185-5p, miR-370-3p, miR-328-3p, miR-769-5p, miR-4306; RP11-136K7.2 could target HSPA8 by regulating miR-338-3p. BAK1 is found that upregulated in NASH and ASH and is involved in the development of NASH/ASH into hepatocellular carcinoma (Nguyen et al., 2018). Moreover, HSPA8 participates in lipolysis by interacting with PLIN2(Kaushik and Cuervo, 2016).

Herein, we reported a hitherto undocumented association between autophagy and lncRNAs in NAFLD and established putative autophagy-related ceRNA networks. However, this study still has limitations. First, we only validated autophagy-related lncRNAs and autophagy-related genes, while miRNAs were not validated. Besides, *in vitro* and *in vivo* experiments were not performed to verify the functions of lncRNAs and their ceRNA networks. Finally, due to the lack of sufficient clinical data, we did not analyze the impact of lncRNAs on the prognosis of patients.

## Conclusion

In this study, four autophagy-related lncRNAs were identified in NAFLD. These lncRNAs and their dominant ceRNA networks deepen our understanding of NAFLD and provide the foothold for future research on NAFLD.

## Data Availability

The original contributions presented in the study are included in the article/supplementary material further inquiries can be directed to the corresponding author.

## References

[B1] Buzzetti E. Pinzani M. Tsochatzis E. A. (2016). The Multiple-Hit Pathogenesis of Non-alcoholic Fatty Liver Disease (NAFLD). Metabolism 65 (8), 1038–1048. 10.1016/j.metabol.2015.12.012 26823198

[B2] Chen Y. Chen X. Gao J. Xu C. Xu P. Li Y. (2019). Long Noncoding RNA FLRL2 Alleviated Nonalcoholic Fatty Liver Disease through Arntl‐Sirt1 Pathway. FASEB J. 33 (10), 11411–11419. 10.1096/fj.201900643rrr 31311301

[B3] Chen L. Wang G. Xu Z. Lin K. Mu S. Pan Y. (2020). Overexpression of LncRNA PSMG3-AS1 Distinguishes Glioblastomas from Sarcoidosis. J. Mol. Neurosci. 70 (12), 2015–2019. 10.1007/s12031-020-01605-9 32529538

[B4] Chen S. Li X. Zhang J. Li L. Wang X. Zhu Y. (2021). Six Mutator-Derived lncRNA Signature of Genome Instability for Predicting the Clinical Outcome of Colon Cancer. J. Gastrointest. Oncol. 12 (5), 2157–2171. 10.21037/jgo-21-494 34790382 PMC8576217

[B5] Chen L.-L. (2016). Linking Long Noncoding RNA Localization and Function. Trends Biochem. Sci. 41 (9), 761–772. 10.1016/j.tibs.2016.07.003 27499234

[B6] Chu Q. Zhang S. Chen M. Han W. Jia R. Chen W. (2019). Cherry Anthocyanins Regulate NAFLD by Promoting Autophagy Pathway. Oxid. Med. Cell Longev. 2019, 4825949. 10.1155/2019/4825949 30931080 PMC6410467

[B7] Cui Y. Fan Y. Zhao G. Zhang Q. Bao Y. Cui Y. (2020). Novel lncRNA PSMG3 AS1 Functions as a miR 143 3p Sponge to Increase the Proliferation and Migration of Breast Cancer Cells. Oncol. Rep. 43 (1), 229–239. 10.3892/or.2019.7390 31661146 PMC6908943

[B8] Di Mauro S. Salomone F. Scamporrino A. Filippello A. Morisco F. Guido M. (2021). Coffee Restores Expression of lncRNAs Involved in Steatosis and Fibrosis in a Mouse Model of NAFLD. Nutrients 13 (9), 2952. 10.3390/nu13092952 34578828 PMC8467439

[B9] Eslam M. Valenti L. Romeo S. (2018). Genetics and Epigenetics of NAFLD and NASH: Clinical Impact. J. Hepatol. 68 (2), 268–279. 10.1016/j.jhep.2017.09.003 29122391

[B10] Ezquerro S. Méndez-Giménez L. Becerril S. Moncada R. Valentí V. Catalán V. (2016). Acylated and Desacyl Ghrelin Are Associated with Hepatic Lipogenesis, β-oxidation and Autophagy: Role in NAFLD Amelioration after Sleeve Gastrectomy in Obese Rats. Sci. Rep. 6, 39942. 10.1038/srep39942 28008992 PMC5180230

[B11] Fatica A. Bozzoni I. (2014). Long Non-coding RNAs: New Players in Cell Differentiation and Development. Nat. Rev. Genet. 15 (1), 7–21. 10.1038/nrg3606 24296535

[B12] Friedman S. L. Neuschwander-Tetri B. A. Rinella M. Sanyal A. J. (2018). Mechanisms of NAFLD Development and Therapeutic Strategies. Nat. Med. 24 (7), 908–922. 10.1038/s41591-018-0104-9 29967350 PMC6553468

[B13] Glick D. Barth S. Macleod K. F. (2010). Autophagy: Cellular and Molecular Mechanisms. J. Pathol. 221 (1), 3–12. 10.1002/path.2697 20225336 PMC2990190

[B14] González-Rodríguez Á. Mayoral R. Agra N. Valdecantos M. P. Pardo V. Miquilena-Colina M. E. (2014). Impaired Autophagic Flux Is Associated with Increased Endoplasmic Reticulum Stress during the Development of NAFLD. Cell Death Dis. 5 (4), e1179. 10.1038/cddis.2014.162 24743734 PMC4001315

[B15] Guo C. Li X. Xie J. Liu D. Geng J. Ye L. (2021). Long Noncoding RNA SNHG1 Activates Autophagy and Promotes Cell Invasion in Bladder Cancer. Front. Oncol. 11, 660551. 10.3389/fonc.2021.660551 34055628 PMC8158816

[B16] Gupta R. Ambasta R. K. Pravir Kumar K. (2021). Autophagy and Apoptosis Cascade: Which Is More Prominent in Neuronal Death? Cell. Mol. Life Sci. 78 (24), 8001–8047. 10.1007/s00018-021-04004-4 34741624 PMC11072037

[B17] Houben T. Oligschlaeger Y. Hendrikx T. Bitorina A. V. Walenbergh S. M. A. van Gorp P. J. (2017). Cathepsin D Regulates Lipid Metabolism in Murine Steatohepatitis. Sci. Rep. 7 (1), 3494. 10.1038/s41598-017-03796-5 28615690 PMC5471235

[B18] Jin E. Huang C. Zhang L. Chen S. Zhao X. Ren Z. (2021). Expression of Oncogenic Long Noncoding RNA PSMG3-antisense 1 in Lung Squamous Cell Carcinoma. Oncol. Lett. 22 (5), 751. 10.3892/ol.2021.13012 34539855 PMC8436406

[B19] Kaushik S. Cuervo A. M. (2016). AMPK-dependent Phosphorylation of Lipid Droplet Protein PLIN2 Triggers its Degradation by CMA. Autophagy 12 (2), 432–438. 10.1080/15548627.2015.1124226 26902588 PMC4835968

[B20] Kim Y. S. Kim S. G. (2020). Endoplasmic Reticulum Stress and Autophagy Dysregulation in Alcoholic and Non-alcoholic Liver Diseases. Clin. Mol. Hepatol. 26 (4), 715–727. 10.3350/cmh.2020.0173 32951410 PMC7641579

[B21] Klionsky D. J. Emr S. D. (2000). Autophagy as a Regulated Pathway of Cellular Degradation. Science 290 (5497), 1717–1721. 10.1126/science.290.5497.1717 11099404 PMC2732363

[B22] Klionsky D. J. Petroni G. Amaravadi R. K. Baehrecke E. H. Ballabio A. Boya P. (2021). Autophagy in Major Human Diseases. EMBO J. 40 (19), e108863. 10.15252/embj.2021108863 34459017 PMC8488577

[B23] Lebeaupin C. Vallée D. Rousseau D. Patouraux S. Bonnafous S. Adam G. (2018). Bax Inhibitor-1 Protects from Nonalcoholic Steatohepatitis by Limiting Inositol-Requiring Enzyme 1 Alpha Signaling in Mice. Hepatology 68 (2), 515–532. 10.1002/hep.29847 29457838

[B24] Li J. Chen X. Kang R. Zeh H. Klionsky D. J. Tang D. (2021). Regulation and Function of Autophagy in Pancreatic Cancer. Autophagy 17 (11), 3275–3296. 10.1080/15548627.2020.1847462 33161807 PMC8632104

[B25] Liang H. Su X. Wu Q. Shan H. Lv L. Yu T. (2020). LncRNA 2810403D21Rik/Mirf Promotes Ischemic Myocardial Injury by Regulating Autophagy through Targeting Mir26a. Autophagy 16 (6), 1077–1091. 10.1080/15548627.2019.1659610 31512556 PMC7469676

[B26] Lin X. Lim I. Y. Lim I. Y. Wu Y. Teh A. L. Chen L. (2017). Developmental Pathways to Adiposity Begin before Birth and are Influenced by Genotype, Prenatal Environment and Epigenome. BMC Med. 15 (1), 50. 10.1186/s12916-017-0800-1 28264723 PMC5340003

[B27] Liu C.-y. Stücker I. Chen C. Goodman G. McHugh M. K. D'Amelio A. M. (2015). Genome-wide Gene-Asbestos Exposure Interaction Association Study Identifies a Common Susceptibility Variant on 22q13.31 Associated with Lung Cancer Risk. Cancer Epidemiol. Biomarkers Prev. 24 (10), 1564–1573. 10.1158/1055-9965.epi-15-0021 26199339 PMC4592421

[B28] Liu B. Deng X. Jiang Q. Li G. Zhang J. Zhang N. (2020). Scoparone Improves Hepatic Inflammation and Autophagy in Mice with Nonalcoholic Steatohepatitis by Regulating the ROS/P38/Nrf2 axis and PI3K/AKT/mTOR Pathway in Macrophages. Biomed. Pharmacother. 125, 109895. 10.1016/j.biopha.2020.109895 32000066

[B29] Lonardo A. Bellentani S. Argo C. K. Ballestri S. Byrne C. D. Caldwell S. H. (2015). Epidemiological Modifiers of Non-alcoholic Fatty Liver Disease: Focus on High-Risk Groups. Dig. Liver Dis. 47 (12), 997–1006. 10.1016/j.dld.2015.08.004 26454786

[B30] Lum J. J. DeBerardinis R. J. Thompson C. B. (2005). Autophagy in Metazoans: Cell Survival in the Land of Plenty. Nat. Rev. Mol. Cell Biol. 6 (6), 439–448. 10.1038/nrm1660 15928708

[B31] Man S. Li X. Zhu W. (2021). miR-4417 Targets lncRNA PSMG3-AS1 to Suppress Cell Invasion and Migration in Cervical Squamous Cell Carcinoma. Oncol. Lett. 22 (1), 502. 10.3892/ol.2021.12763 33986863 PMC8114464

[B32] Marrero J. A. Fontana R. J. Su G. L. Conjeevaram H. S. Emick D. M. Lok A. S. (2002). NAFLD May Be a Common Underlying Liver Disease in Patients with Hepatocellular Carcinoma in the United States. Hepatology 36 (6), 1349–1354. 10.1002/hep.1840360609 12447858

[B33] Nguyen L. Masouminia M. Mendoza A. Samadzadeh S. Tillman B. Morgan T. (2018). Alcoholic Hepatitis versus Non-alcoholic Steatohepatitis: Levels of Expression of Some Proteins Involved in Tumorigenesis. Exp. Mol. Pathology 104 (1), 45–49. 10.1016/j.yexmp.2017.12.007 PMC5800980 29307797

[B34] Ren H. Guo X. Li F. Xia Q. Chen Z. Xing Y. (2021). Four Autophagy-Related Long Noncoding RNAs Provide Coexpression and ceRNA Mechanisms in Retinoblastoma through Bioinformatics and Experimental Evidence. ACS Omega 6 (49), 33976–33984. 10.1021/acsomega.1c05259 34926945 PMC8674985

[B35] Rohilla S. Awasthi A. Kaur S. Puria R. (2021). Evolutionary Conservation of Long Non-coding RNAs in Non-alcoholic Fatty Liver Disease. Life Sci. 264, 118560. 10.1016/j.lfs.2020.118560 33045214

[B36] Salmena L. Poliseno L. Tay Y. Kats L. Pandolfi P. P. (2011). A ceRNA Hypothesis: The Rosetta Stone of a Hidden RNA Language? Cell 146 (3), 353–358. 10.1016/j.cell.2011.07.014 21802130 PMC3235919

[B37] Savary S. Trompier D. Andréoletti P. Le Borgne F. Demarquoy J. Lizard G. (2012). Fatty Acids - Induced Lipotoxicity and Inflammation. Curr. Drug Metab. 13 (10), 1358–1370. 10.2174/138920012803762729 22978392

[B38] Singh R. Kaushik S. Wang Y. Xiang Y. Novak I. Komatsu M. (2009). Autophagy Regulates Lipid Metabolism. Nature 458 (7242), 1131–1135. 10.1038/nature07976 19339967 PMC2676208

[B39] Statello L. Guo C.-J. Chen L.-L. Huarte M. (2021). Gene Regulation by Long Non-coding RNAs and its Biological Functions. Nat. Rev. Mol. Cell Biol. 22 (2), 96–118. 10.1038/s41580-020-00315-9 33353982 PMC7754182

[B40] Ueno T. Komatsu M. (2017). Autophagy in the Liver: Functions in Health and Disease. Nat. Rev. Gastroenterol. Hepatol. 14 (3), 170–184. 10.1038/nrgastro.2016.185 28053338

[B41] Waeber G. Delplanque J. Bonny C. Mooser V. Steinmann M. Widmann C. (2000). The Gene MAPK8IP1, Encoding Islet-Brain-1, is a Candidate for Type 2 Diabetes. Nat. Genet. 24 (3), 291–295. 10.1038/73523 10700186

[B42] Wang P.-X. Ji Y.-X. Zhang X.-J. Zhao L.-P. Yan Z.-Z. Zhang P. (2017). Targeting CASP8 and FADD-like Apoptosis Regulator Ameliorates Nonalcoholic Steatohepatitis in Mice and Nonhuman Primates. Nat. Med. 23 (4), 439–449. 10.1038/nm.4290 28218919

[B43] Wong C.-M. Tsang F. H.-C. Ng I. O.-L. (2018). Non-coding RNAs in Hepatocellular Carcinoma: Molecular Functions and Pathological Implications. Nat. Rev. Gastroenterol. Hepatol. 15 (3), 137–151. 10.1038/nrgastro.2017.169 29317776

[B44] Xia Y. Zhang Y. Wang H. (2021a). Upregulated lncRNA HCG18 in Patients with Non-alcoholic Fatty Liver Disease and its Regulatory Effect on Insulin Resistance. Diabetes Metab. Syndr. Obes. 14, 4747–4756. 10.2147/dmso.s333431 34887672 PMC8651094

[B45] Xia Y. Zhen L. Li H. Wang S. Chen S. Wang C. (2021b). MIRLET7BHG Promotes Hepatocellular Carcinoma Progression by Activating Hepatic Stellate Cells through Exosomal SMO to Trigger Hedgehog Pathway. Cell Death Dis. 12 (4), 326. 10.1038/s41419-021-03494-1 33771969 PMC7997896

[B46] Xiong H. Shen J. Chen Z. Yang J. Xie B. Jia Y. (2020). H19/let-7/Lin28 ceRNA Network Mediates Autophagy Inhibiting Epithelial-mesenchymal Transition in Breast Cancer. Int. J. Oncol. 56 (3), 794–806. 10.3892/ijo.2020.4967 32124962

[B47] Yang L. Li P. Fu S. Calay E. S. Hotamisligil G. S. (2010). Defective Hepatic Autophagy in Obesity Promotes ER Stress and Causes Insulin Resistance. Cell Metab. 11 (6), 467–478. 10.1016/j.cmet.2010.04.005 20519119 PMC2881480

[B48] Yue N. Ye M. Zhang R. Guo Y. (2020). MiR-449b-5p Targets lncRNA PSMG3-AS1 to Suppress Cancer Cell Proliferation in Lung Adenocarcinoma. BMC Pulm. Med. 20 (1), 152. 10.1186/s12890-020-01189-5 32471413 PMC7260832

[B49] Zhang J. Huang J. Chen W. Hu Z. Wang X. (2020). miR-143-3p Targets lncRNA PSMG3-AS1 to Inhibit the Proliferation of Hepatocellular Carcinoma Cells. Cancer Manag. Res. 12, 6303–6309. 10.2147/cmar.s242179 32801875 PMC7394512

[B50] Zhao X.-Y. Xiong X. Liu T. Mi L. Peng X. Rui C. (2018). Long Noncoding RNA Licensing of Obesity-Linked Hepatic Lipogenesis and NAFLD Pathogenesis. Nat. Commun. 9 (1), 2986. 10.1038/s41467-018-05383-2 30061575 PMC6065308

[B51] Zhao C. Wang Y. Tu F. Zhao S. Ye X. Liu J. (2021). A Prognostic Autophagy-Related Long Non-coding RNA (ARlncRNA) Signature in Acute Myeloid Leukemia (AML). Front. Genet. 12, 681867. 10.3389/fgene.2021.681867 34276784 PMC8278057

[B52] Zhou J. Zhou F. Wang W. Zhang X. J. Ji Y. X. Zhang P. (2020). Epidemiological Features of NAFLD from 1999 to 2018 in China. Hepatology 71 (5), 1851–1864. 10.1002/hep.31150 32012320

[B53] Zhou X. Xu S.-N. Yuan S.-T. Lei X. Sun X. Xing L. (2021). Multiple Functions of Autophagy in Vascular Calcification. Cell Biosci. 11 (1), 159. 10.1186/s13578-021-00639-9 34399835 PMC8369777

[B54] Zottel A. Šamec N. Videtič Paska A. Jovčevska I. (2020). Coding of Glioblastoma Progression and Therapy Resistance through Long Noncoding RNAs. Cancers (Basel) 12 (7), 1842. 10.3390/cancers12071842 PMC7409010 32650527

